# Genome-wide exploration of a pyroptosis-related gene module along with immune cell infiltration patterns in bronchopulmonary dysplasia

**DOI:** 10.3389/fgene.2022.1074723

**Published:** 2023-01-04

**Authors:** Leiming Chen, Chaofan Shi, Guoping Zhou, Xiaofeng Yang, Zhenqin Xiong, Xiaoxue Ma, Lan Zhu, Xuejiao Ma, Yan Mao, Yifang Hu, Jimei Wang, Xinfang Tang, Yunlei Bao, Yunxia Ma, Fei Luo, Chuyan Wu, Feng Jiang

**Affiliations:** ^1^ Department of Laboratory Medicine, Obstetrics and Gynecology Hospital of Fudan University, Shanghai, China; ^2^ Department of Radiology, Yongping County People’s Hospital, Dali, China; ^3^ Department of Neonatology, Yongping County People’s Hospital, Dali, China; ^4^ Department of Pediatrics, Dali Bai Autonomous Prefecture People’s Hospital, Dali, China; ^5^ Department of Pediatrics, The First Affiliated Hospital of Nanjing Medical University, Nanjing, China; ^6^ Department of Geriatric Endocrinology, The First Affiliated Hospital of Nanjing Medical University, Nanjing, China; ^7^ Department of Neonatology, Obstetrics and Gynecology Hospital of Fudan University, Shanghai, China; ^8^ Department of Nephrology, The Affiliated Lianyungang Oriental Hospital of Xuzhou Medical University, The Affiliated Lianyungang Oriental Hospital of Kangda College of Nanjing Medical University, The Affiliated Lianyungang Oriental Hospital of Bengbu Medical College, Lianyungang, China; ^9^ Department of Rehabilitation Medicine, The First Affiliated Hospital of Nanjing Medical University, Nanjing, China

**Keywords:** bioinformatics, bronchopulmonary dysplasia, diagnostic, pyroptosis, immune microenvironment

## Abstract

Pyroptosis plays a crucial role in bronchopulmonary dysplasia (BPD) and is associated with various lung injury illnesses. However, the function of pyroptosis-related genes (PRGs) in BPD remains poorly understood. The gene expression omnibus (GEO) database was searched for information on genes associated with BPD. Twenty-five BPD-related DE-PRGs were identified, all of which were closely associated with pyroptosis regulation and immunological response. LASSO and SVM-RFE algorithms identified CHMP7, NLRC4, NLRP2, NLRP6, and NLRP9 among the 25 differentially expressed PRGs as marker genes with acceptable diagnostic capabilities. Using these five genes, we also generated a nomogram with excellent predictive power. Annotation enrichment analyses revealed that these five genes may be implicated in BPD and numerous BPD-related pathways. In addition, the ceRNA network showed an intricate regulatory link based on the marker genes. In addition, CIBERSORT-based studies revealed that alterations in the immunological microenvironment of BPD patients may be associated with the marker genes. We constructed a diagnostic nomogram and gave insight into the mechanism of BPD. Its diagnostic value for BPD must be evaluated in further research before it can be used in clinical practice.

## Introduction

Bronchopulmonary dysplasia (BPD) is the leading chronic respiratory disease in preterm newborns and is associated with a high mortality rate (Gilfillan et al., 2021). BPD is caused by prenatal and perinatal conditions that impair lung development in very premature newborns. With developments in perinatal care, the proportion of preterm newborns successfully treated with BPD has increased. Preterm newborns have exceedingly immature organs, and respiratory insufficiency continues to be the leading cause of prenatal morbidity and mortality (Gibbs et al., 2020). Since the majority of children with extremely low birth weight are born during the crucial saccular stage of lung development, BPD may result in aberrant lung development and impaired lung function throughout the patient’s lifetime. BPD may also cause harm to the neurological system and other systemic organs (Shukla and Ambalavanan, 2021). However, the pathophysiology of BPD is poorly understood, and there are few early diagnostic tests and precise preventative or therapeutic approaches for this disorder.

Pyroptosis, also known as gasdermin-mediated programmed cell death, is triggered by inflammasomes and induced by the activation of caspase-1 and caspase-4/5/11 in canonical and noncanonical pathways, respectively, resulting in DNA cleavage, cell and organelle swelling, membrane rupture, and release of endocellular proinflammatory cytokines, including IL-1 and IL-18 (Kovacs and Miao, 2017; Shi et al., 2017; Yu et al., 2021). Pyroptosis may also prevent infection and promote adaptive immune responses (Kesavardhana et al., 2020). Pyroptosis generally protects multicellular host organisms from invading pathogens; nonetheless, its overactivation may result in septic shock and sepsis (Zheng et al., 2021). Apoptosis, a kind of programmed cell death, is regulated by the equilibrium between proapoptotic and prosurvival B-cell lymphoma (BCL)-2 proteins. Apoptosis may be transformed into pyroptosis in response to an invading pathogen (Bertheloot et al., 2021). Apoptosis, a programmed cell death, is widely regarded to be harmless. Several studies have shown that pyroptosis plays a variety of functions in numerous illnesses, including disorders of the neurological system, autoimmune diseases, infectious diseases, cardiovascular diseases, and cancers (Xia et al., 2019; Hou et al., 2021; Ji et al., 2021).

Increasing evidence suggests that pyroptosis is essential for inflammation-related respiratory disorders, such as acute lung damage, chronic obstructive pulmonary disease, and asthma, and pyroptosis has been directly linked to the pathogenesis of BPD (Tsai et al., 2018; Zhuang et al., 2020). Zou et al. discovered that the decrease in maternally expressed gene 3 (MEG3) suppresses NLRP3 inflammasome and caspase-1 signaling to reduce pyroptosis and relieve hyperoxia-induced lung damage (Zou et al., 2020). Pyroptosis of alveolar macrophages may contribute to the development of ALI *via* the mechanism of mediating inflammation (Cheng et al., 2021; Jiao et al., 2021). It has also been observed that gasdermin D (GSDMD)-dependent pyroptosis is induced by lipopolysaccharide (LPS) in alveolar macrophages *via* TLR4/NLRP3/caspase-1 pathway activation, leading to severe lung injury defined by infiltration of inflammatory cells (Jiang et al., 2021; Liu et al., 2022).

Resultantly, a machine learning algorithm was used to analyze and validate the accuracy of using pyroptosis-related genes (PRGs) as biomarkers for BPD. Additionally, bioinformatics was used to delve into the roles of these genes in the immune microenvironment. This study provides new insight into the pathophysiology of BPD as well as the development of therapeutic interventions for patients who suffer from this condition.

## Materials and methods

### Collection and processing of data

We retrieved the gene expression data for BPD and control samples from the Gene Expression Omnibus (GEO) database. GSE32472 provides microarray profiles of blood samples from neonates with BPD, including microarray analyses of gene expression on the 5th, 14th, and 28th days of life. On day 28, after a more definitive diagnosis of BPD was established, 100 blood samples were chosen at random to verify the reliability of the selection process. These samples were collected from 38 controls and 62 patients with BPD. We calculated the Effect size d to evaluate the statistical power of the sample size *via* G*Power software (Faul et al., 2009), which confirmed the sample size was reasonable. The gene set enrichment analysis (GSEA) public database was searched with the term “pyroptosis.” Two gene sets were obtained, GOBP PYROPTOSIS and REACTOME PYROPTOSIS. In total, 40 PRGs were discovered. In addition, 33 PRGs were identified based on previous reports. Finally, 57 PRGs were identified (Supplementary Table S1).

### Differential expression analysis

Expression data for 55 PRGs were initially retrieved from the GSE32472 dataset for control and BPD samples. Afterward, we run the Wilcoxon test in the R software to identify the differentially expressed PRGs (DE-PRGs) between the two samples. A *p*-value less than 0.05 was chosen to serve as the criterion for statistical significance.

### Annotation enrichment analysis

The Metascape database (http://metascape.org) was employed to explore the biological significance of the differentially expressed genes (Zhou et al., 2019). Gene Ontology (GO) and Kyoto Encyclopedia of Genomes (KEGG) pathway enrichment analyses were performed using specific genes. The minimum overlap required for statistical significance was set at three, and the *p*-value was less than 0.01.

### Optimal BPD diagnostic gene biomarker identification

With the aid of the glmnet package, the LASSO algorithm was applied to the data to minimize its dimensionality. To select markers and identify gene biomarkers for BPD, the DE-PRGs between control and BPD samples were retained. The SVM package in R was used to develop support vector machine-recursive feature elimination (SVM-RFE) models, and the average misjudgment rates of their 10-fold cross-validation were compared. In addition, optimum gene biomarkers for BPD were discovered using biomarkers produced from both algorithms that overlapped. The diagnostic capabilities of the optimal gene biomarkers were evaluated by generating the receiver operating characteristic (ROC) curve and determining the area under the curve (AUC), sensitivity, specificity, and accuracy. Also, the prediction function in the R package was used for the purpose of developing a logistic regression model based on the five marker genes. This was carried out to predict the types of samples present in the GSE32472 dataset. We used ROC curves to assess the logistic regression model’s diagnostic power.

### Single-gene GSEA

The GSEA R package was used to perform this analysis. To further investigate the linked pathways of the five marker genes, the correlation between these marker genes and the other genes in the GSE32472 dataset was evaluated. The genes were then ranked, from highest to lowest, based on their correlations, and the ranked genes were referred to as the test set. The KEGG pathway set served as a predetermined set to discover its enrichment in the gene set.

### Single-gene gene set variation analysis (GSVA)

The GSVA R package was used for this analysis. The KEGG pathway enrichment set was used as the background gene set to analyze each marker gene. Using the limma program, we simultaneously performed an analysis of the difference in GSVA scores that existed between groups that had high and low-expression levels of the marker genes. The screening condition difference were *p* < 0.05 and |t| > 2. When t was more than zero, the pathway was considered to be enriched in the group that had high expression levels; conversely, when t was less than zero, the pathway was considered enriched in the group that had low expression levels.

### Immune infiltration analysis

CIBERSORT is a technique for determining the cell fraction of complex tissues based on gene expression profiles (Newman et al., 2015). We used CIBERSORT to predict the proportion of 22 different types of infiltrating immune cells in each sample from the GSE32472 dataset. All immune cell type fractions were summed up to one for each sample.

### Establishment of competing endogenous RNA (ceRNA) network

mRNA–miRNA interaction pairs based on the five marker genes were predicted using the starBase database (Li et al., 2014). RNA sequences for the five marker genes were retrieved from the NCBI website, while human miRNA sequences were retrieved from the miRBase database. mRNA–miRNA nucleic acid binding was predicted using miRanda, and the binding score cutoff value was elevated from 140 to 170. The predicted miRNA was then searched in starBase and miRNA-lncRNAs were screened to obtain the ceRNA network of mRNA-miRNA-lncRNA.

### Establishment of nomogram

A diagnostic nomogram based on selected candidate PRGs was established using the “rms” package in the R software. The consistency between our predicted values and reality was assessed using a calibration curve. Clinical impact curve analysis and decision curve analysis (DCA) were carried out to determine whether or not the decisions made based on the model were favorable to the patients.

### *In vitro* experiments

The JCRB Cell Bank provided the human lung adenocarcinoma epithelial cell line, A549. It was grown in DMEM with 1% penicillin/streptomycin solution and 10% fetal bovine serum (Thermo Fisher Science, Waltham, MA). Experiments on hyperoxia were carried out in a Modular Incubator Chamber (MIC-101, Billirups-Rothenberg Inc., Del-Mar, CA) in accordance with the manufacturer’s instructions. In details, the hyperoxia group was cultivated in a MIC-101 incubator with 85% O2 and 5% CO2, whereas the normoxia (NO) group continued to be cultured in an incubator with 21% O2 and 5% CO2. The RNeasy Mini Kit was used to extract total RNA (Qiagen, Hilden, Germany). Utilizing ReverTra Ace, RNA was reverse transcribed into cDNA (Toyobo, Osaka, Japan). Using the LightCycler 480 (Roche Applied Science, Indianapolis, IN), quantitative real-time PCR was carried out using SYBR Premix Ex Taq II (Takara Bio, Shiga, Japan). Primers used were designed in PrimerBank (http://pga.mgh.harvard.edu/primerbank/) and synthesized by Generay (Shanghai, China). The sequences were as follows: NLRC4, forward 5′-TGC​ATC​ATT​GAA​GGG​GAA​TCT​G-3′ and reverse 5′- GAT​TGT​GCC​AGG​TAT​ATC​CAG​G-3′; CHMP7, forward 5′- AAG​CCT​CTC​AAG​TGG​ACT​CTT-3′ and reverse 5′- ACA​GAC​GAT​ACA​CCT​CCT​CAG-3′; NLRP2, forward 5′- TGG​CCT​GGA​GAT​AGC​AAA​GAG -3′ and reverse 5′- CAC​CAC​CGT​GTA​TGA​GAA​GGG -3′; NLRP6, forward 5′- CCT​ACC​AGT​TCA​TCG​ACC​AGA-3′ and reverse 5′- CTC​AGC​AGT​CCG​AAG​AGG​AA -3′; NLRP9, forward 5′- TTT​GGC​TTG​TTG​TGG​TAT​CTG​AA -3′ and reverse 5′- CTG​GGT​AAT​GTT​TGT​CCA​GCA-3′; and GAPDH, forward 5′- GGA​GCG​AGA​TCC​CTC​CAA​AAT-3′ and reverse 5′- GGC​TGT​TGT​CAT​ACT​TCT​CAT​GG -3′.

### Statistical analysis

The BPD *vs*. control groups were compared by the Wilcoxon test. The relationship between the DE-PRGs was determined using Pearson’s correlation analysis. The Jvenn package was employed to generate the Venn diagrams. The ceRNA network was visualized using Cytoscape version 3.9.1. A *p*-value less than 0.05 was considered significant. The R package was used for all of the analyses that were conducted.

## Results

### Identification of DE-PRGs in GSE32472

**FIGURE 1:**
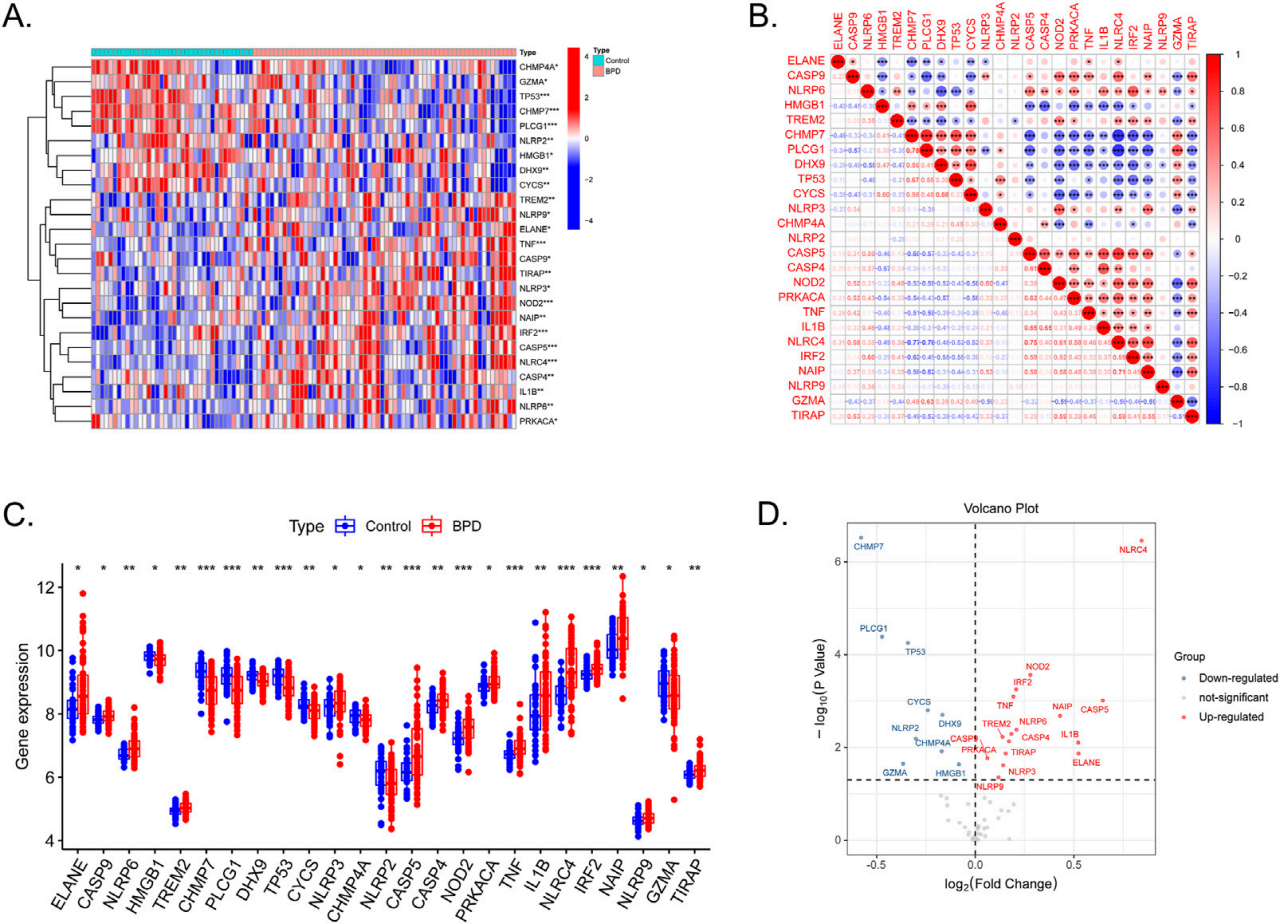
Expression levels of DE-PRGs in BPD. **(A)** Heatmap shows expression patterns of DE-PRGs. **(B)** Correlations between these genes and BPD. **(C)** The expression of the 25 DE-PRGs in BPD and control samples. **(D)** The volcano plot with visualized DE-PRGs.

In total, 25 out of 55 PRGs, including nine downregulated and 16 upregulated genes, were found to be differentially expressed between control and samples in the GSE32472 dataset (Supplementary Table S2). The expression patterns of the DE-PRGs were visualized in a clustering heatmap (Figure 1A). Figure 1B presents the correlation between these genes. As shown in Figure 1C, D, NLRP6, NLRC4, NLRP9 and other 13 DE-PRGs were upregulated in BPD group, while CHMP7, NLRP2, and other 4 DE-PRGs were downregulated, further confirming the results in Figure 1A.

### Annotation enrichment analysis

**FIGURE 2:**
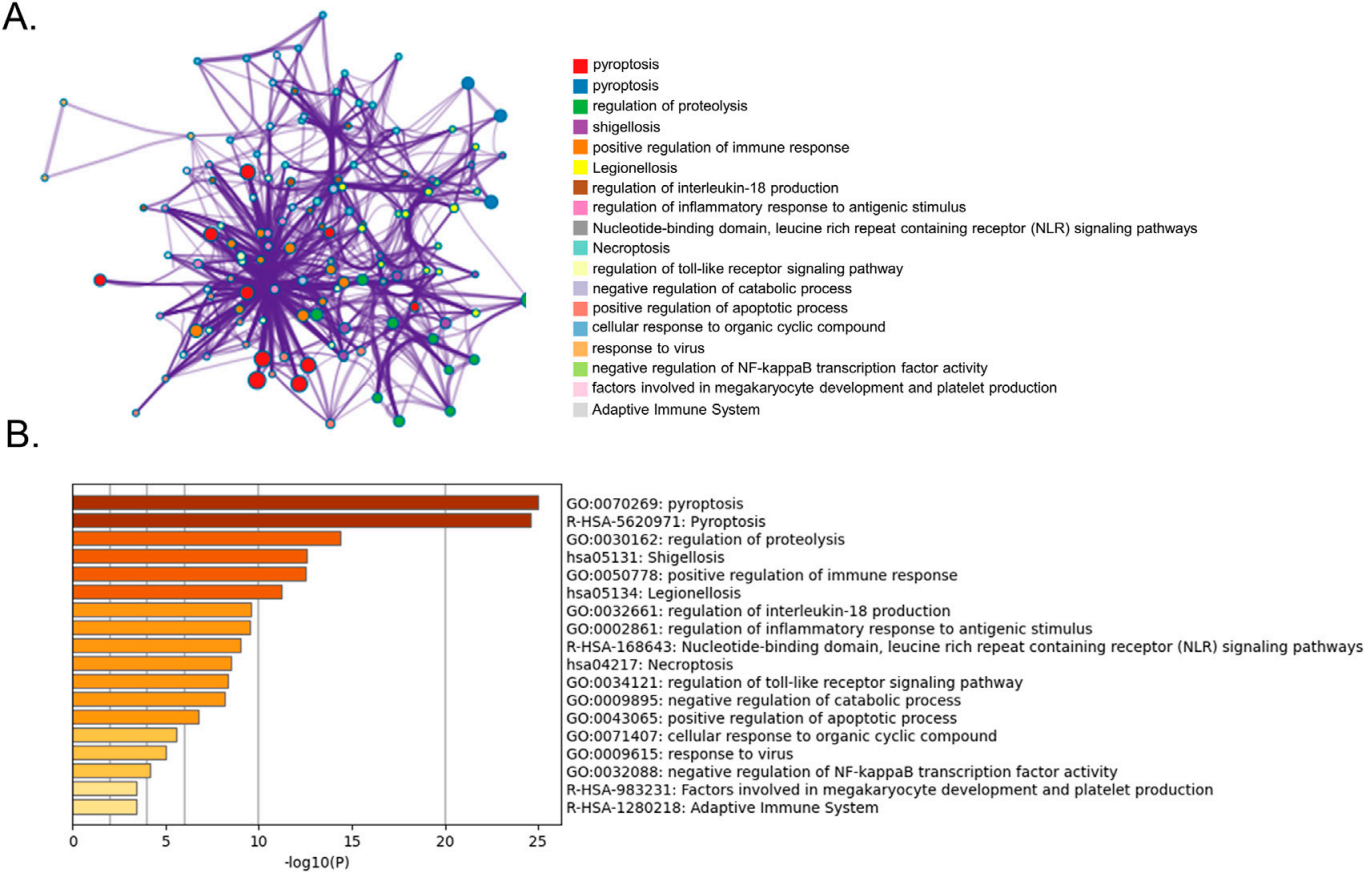
Functional analysis of 25 DE-PRGs. **(A)** Network of enriched terms. **(B)** Bar graph showing biological pathways and their *p*-values.

GO and KEGG pathway enrichment analyses were performed using Metascape. The top GO and KEGG pathways were pyroptosis, regulation of proteolysis, shigellosis, positive immune response regulation, legionellosis, and regulation of interleukin-18 production, indicating that DE-PRGs may be involved in the pathogenesis of BPD (Figure 2).

### Identification of 5 DE-PRGs as diagnostic genes for BPD

**FIGURE 3:**
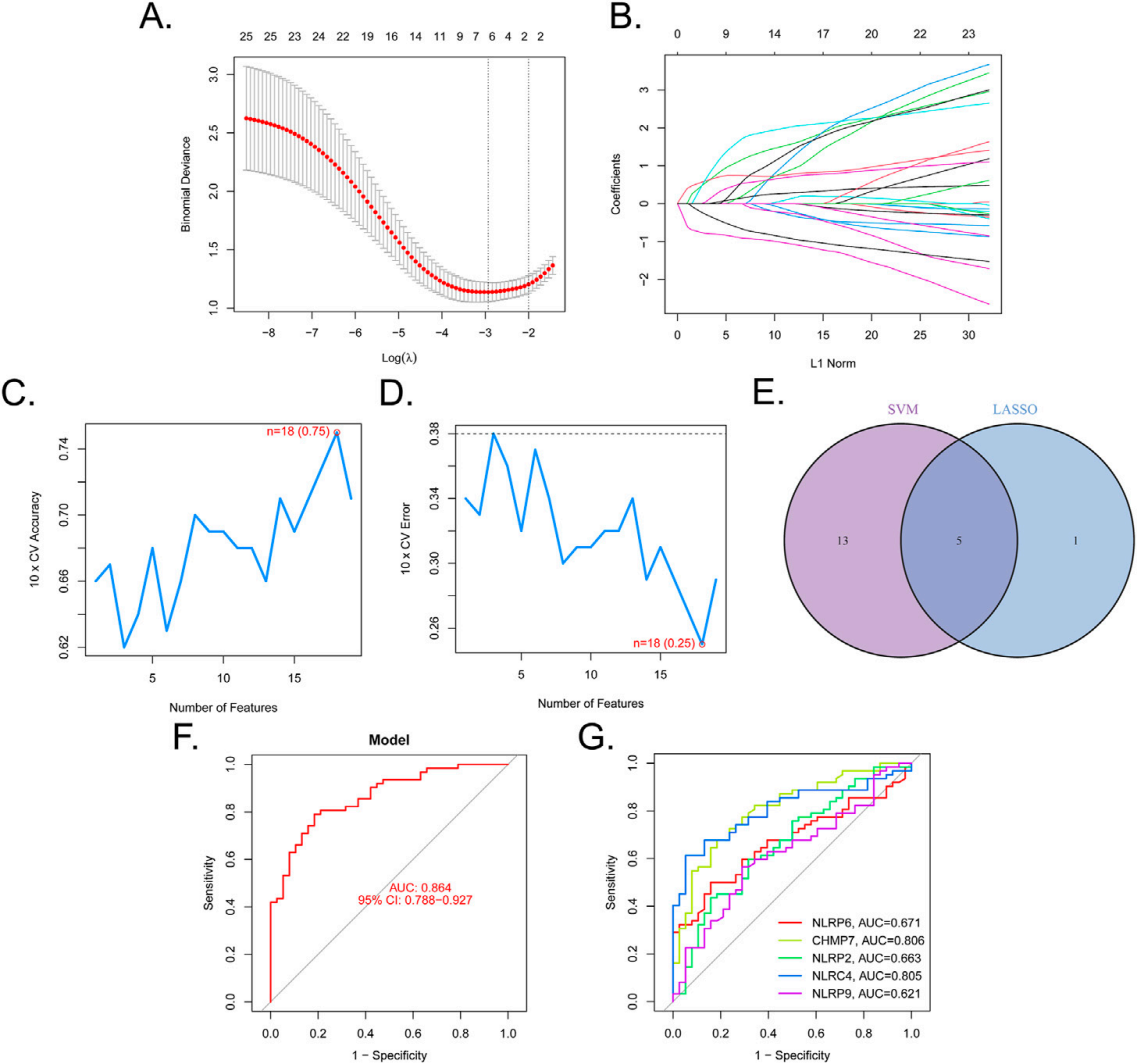
Five DE-PRGs were identified as biomarkers for BPD. **(A,B)** LASSO algorithm with penalty parameter tuning conducted by 10-fold cross-validation was used to select six BPD-related markers. **(C,D)** SVM-RFE algorithm to screen the 25 DE-PRGs to identify the optimal combination of marker genes. In total, 18 genes were identified as the optimal marker genes. **(E)** Marker genes obtained from the LASSO and SVM-RFE models. **(F)** Logistic regression model to determine the AUC of BPD samples. **(G)** ROC curves for the five biomarkers.

We then determined the diagnostic potential of the DE-PRGs. We then used the SVM-RFE and LASSO algorithms to screen the DE-PRGs in the GSE32472 dataset to differentiate BPD from control samples. Six BPD-related markers were selected using the LASSO regression with penalty parameter tuning carried out by 10-fold cross-validation (Figures 3A, B). The 25 DE-PRGs were then filtered using the SVM-RFE algorithm to determine the optimal combination of feature genes. The optimal feature genes were ultimately found to be 18 in number (maximal accuracy = 0.75, minimal RMSE = 0.25) (Figures 3C, D). Five marker genes (NLRP6, CHMP7, NLRP2, NLRC4, and NLRP9) were identified for further analysis after the marker genes were subjected to an intersection (Figure 3E). To further confirm the roles of the five marker genes in BPD, A549 cells were cultured in a special incubator containing 85% O2 and 5% CO2 to simulate the BPD state, while control cells were cultured in 21% O2 and 5% CO2. The RT-qPCR results in Supplementary Figure S1 showed that NLRP6, NLRC4, and NLRP9 were elevated in the hyperoxia group, while CHMP7 and NLRP2 were downregulated. The difference in the expression levels of the five genes between hyperoxia and normoxia groups showed the same trends as those between BPD and control samples.

A regression model based on the five marker genes was established using the glmnet package in R. Following this, ROC curves revealed that the model distinguished between control and BPD samples (AUC = 0.864, Figure 3F). Additionally, ROC curves were used to assess the association between the individual genes and BPD. The AUC was higher than 0.6 for each gene (Figure 3G). These findings suggest the logistic regression model had a higher level of specificity and accuracy than the individual marker genes.

### Establishment of nomogram

**FIGURE 4:**
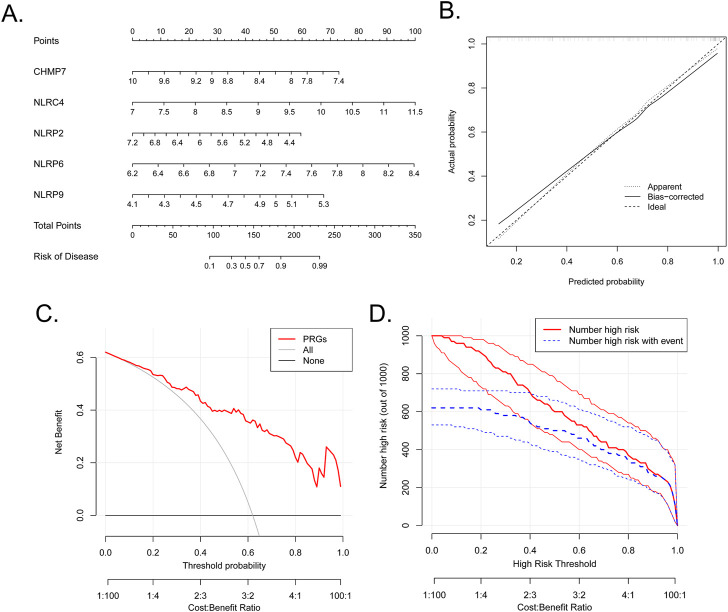
Establishment of the nomogram. **(A)** Establishment of a nomogram based on the five biomarkers. **(B)** Calibration curve revealing the predictive ability of the nomogram. **(C)** The nomogram may be used to make decisions that favor BPD patients. **(D)** Clinical impact curve revealing the clinical impact of the nomogram.

A diagnostic nomogram based on the five marker PRGs was developed (Figure 4A). The nomogram’s predictive accuracy was highlighted using calibration curves (Figure 4B). The line in the DCA curve remained above the gray and black lines from 0 to 1, suggesting that decisions based on the nomogram model may favor patients with BPD (Figure 4C). The clinical impact curve indicated that the nomogram had a remarkable predictive ability (Figure 4D).

### Marker genes were closely associated with various pathways related to BPD

**FIGURE 5:**
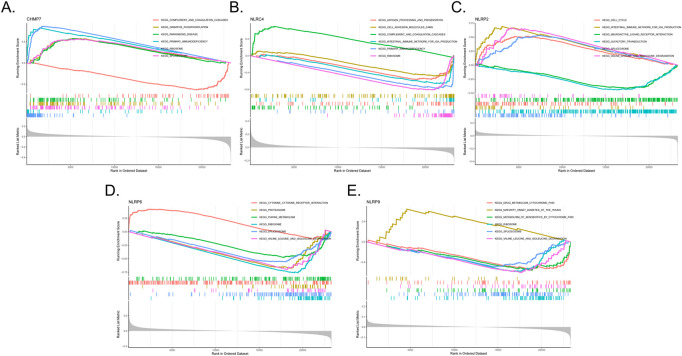
Single-gene GSEA-KEGG pathway analysis in CHMP7 **(A)**, NLRC4 **(B)**, NLRP2 **(C)**, NLRP6 **(D)**, and NLRP9 **(E)**.

Single-gene GSEA-KEGG pathway analysis was carried out to assess the correlation between BPD and the marker genes. Figures 5A–E presents the six leading pathways that were enriched for each of the marker genes. CHMP7 was closely related to complement and coagulation cascades, oxidative phosphorylation, primary immunodeficiency, ribosome, and splicesome. NLRC4 was related to the cell intestinal immune network for IgA production, complement and coagulation cascades, antigen processing and presentation, primary immunodeficiency, adhesion molecules cams, and ribosome. NLRP2 correlated with the intestinal immune network for IgA production, cell cycle, neuroactive ligand–receptor interaction, olfactory transduction, spliceosome, and valine, leucine, and isoleucine degradation. NLRP6 was related to cytokine–cytokine receptor interaction, proteasome, purine metabolism, ribosome, spliceosome, and valine, leucine, and isoleucine degradation. NLRP9 was related to drug metabolism by cytochrome, maturity-onset diabetes of the young, metabolism of xenobiotics by cytochrome, ribosome, spliceosome, and valine, leucine, and isoleucine degradation.

**FIGURE 6:**
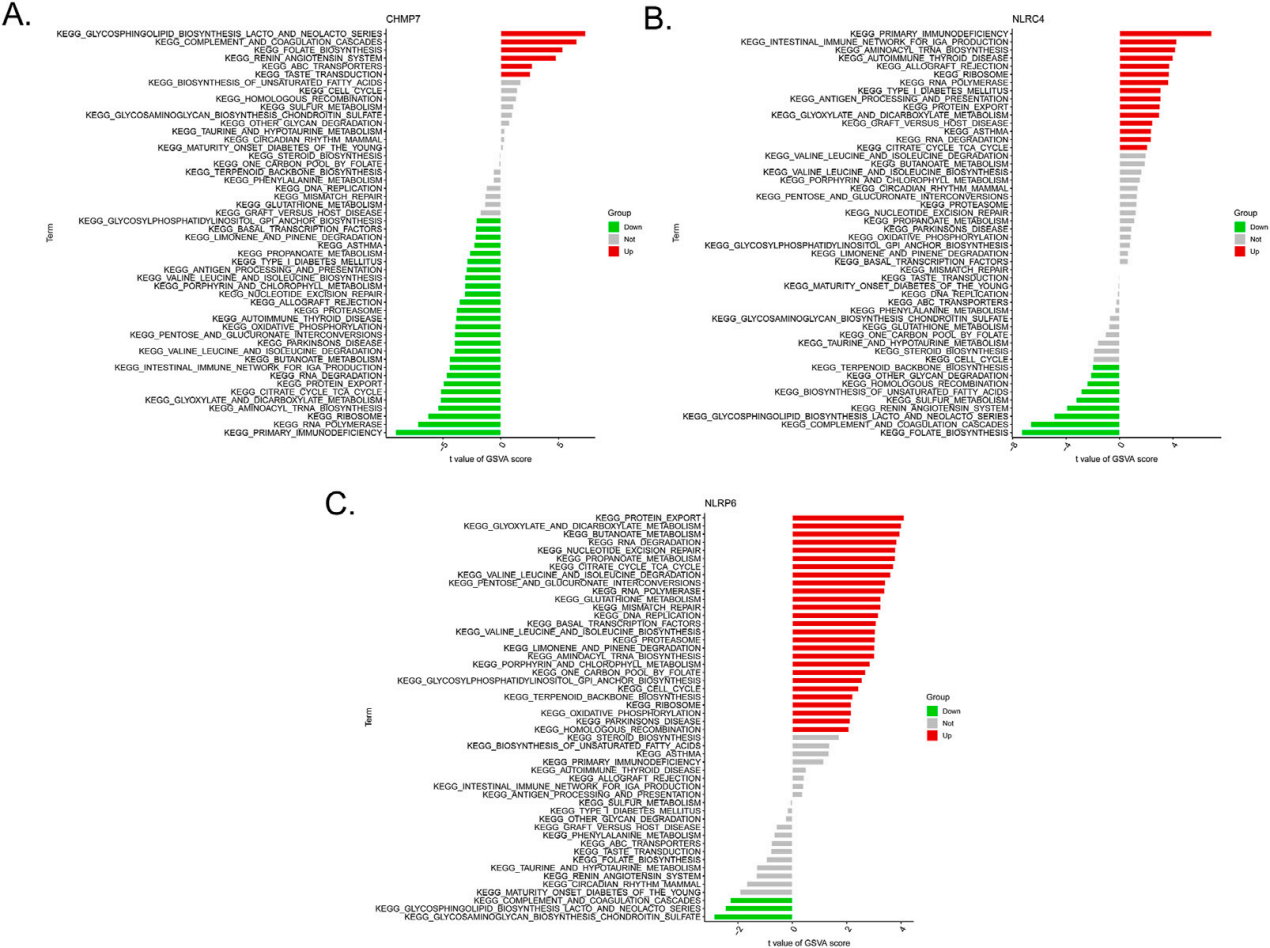
Low- and high-expression groups based on the levels of expression of each marker gene combined with GSVA in CHMP7 **(A)**, NLRC4 **(B)**, and NLRP6 **(C)**.

We then examined the differentially activated pathways between the low- and high-expression groups. Notably, high CHMP7 expression activated glycosphingolipid biosynthesis lacto and neolacto series, complement and coagulation cascades, and folate biosynthesis, whereas the low expression of CHMP7 activated primary immunodeficiency, RNA polymerase, and ribosome. The upregulation of NLRC4 activated the intestinal immune network for IgA production, primary immunodeficiency, and aminoacyl tRNA biosynthesis, whereas the downregulation of NLRC4 activated folate biosynthesis, complement and coagulation cascades, and glycosphingolipid biosynthesis lacto and neolacto series. High NLRP6 expression was associated with protein export, glyoxylate and dicarboxylate metabolism, and butanoate metabolism. Low expression of NLRP6 activated glycosphingolipid biosynthesis lacto and neolacto series, glycosaminoglycan biosynthesis chondroitin sulfate, and complement and coagulation cascades (Figures 6A–C).

### Immune landscape analysis

**FIGURE 7:**
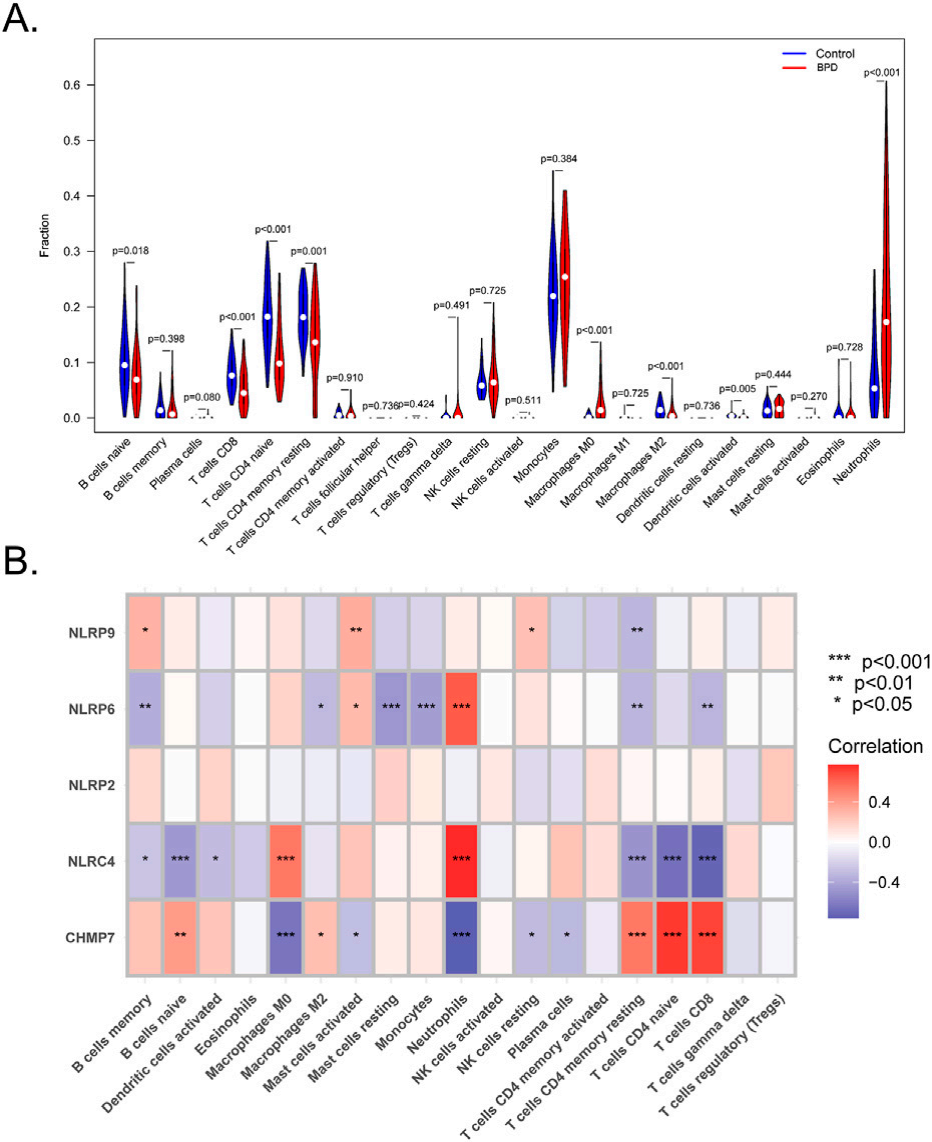
Analysis of the immune landscape. **(A)** CIBERSORT algorithm to evaluate the differences in the immune microenvironment between BPD and control samples. **(B)** Correlations between immune cells and the five marker genes. (^*^ *p* < 0.05, **p < 0.01, ***p < 0.001).

The previous findings suggested an association of the immune response with the marker genes. Furthermore, mounting evidence suggests a close association of BPD with the immune microenvironment. Moreover, the CIBERSORT tool was employed to evaluate the association between the immune microenvironment and BPD. The proportions of neutrophils, macrophages M0, and monocytes were elevated in the BPD samples compared to the control samples, whereas the proportions of T cells CD4 naive, T cells CD4 memory resting, T cells CD8, B cells naïve, macrophages M2, and dendritic cells activated were elevated in the control samples compared to the BPD samples (Figure 7A). Furthermore, Pearson’s correlation analysis indicated remarkable negative and positive correlations between NLRP6 and mast cells resting, monocytes, and neutrophils. Strong negative and positive correlations between NLRC4 and macrophages M0, neutrophils, T cells CD4 naive, T cells CD8, T cells CD4 memory resting, and B cells naive were also observed. Additionally, strong positive and negative correlations between CHMP7 and macrophages M0, neutrophils, T cells CD4 naive, T cells CD8, and T cells CD4 memory resting were revealed (Figure 7B).

### Gene marker-based ceRNA network

**FIGURE 8:**
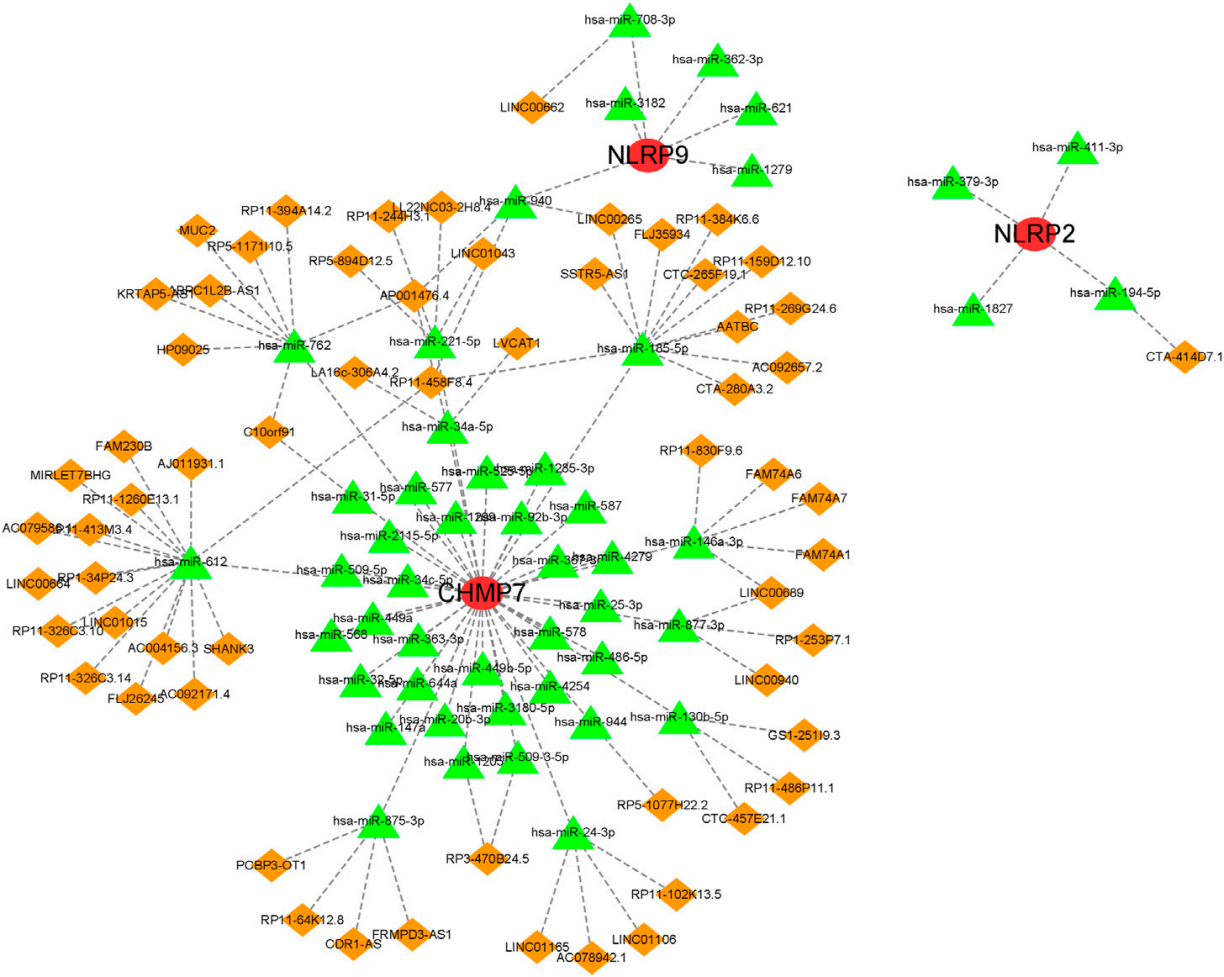
A ceRNA network based on marker genes.

A ceRNA network based on the five marker genes was constructed. A total of 113 nodes (3 marker genes, 48 miRNAs, and 62 lncRNAs) and 118 edges were present in the network (Figure 8). Five lncRNAs regulated the expression of CHMP7 by binding competitively to hsa-miR-146a-3p. However, hsa-miR-185-5p bound to 10 lncRNAs to exhibit its regulatory role on CHMP7. CTA-414D7.1 regulated NLRP2 expression by binding competitively to hsa-miR-194-5p. LINC00662 regulated NLRP9 expression competitively binding to hsa-miR-708-3p.

## Discussion

BPD is a complex condition with a substantial hereditary predisposition. Various pathways and genetic variations, according to some researchers, may be connected with BPD susceptibility. Each genetic variation plays a role in the onset and progression of BPD, and these variants result in the dysregulation of biological processes that occur in the lungs of preterm newborns (Pasha et al., 2018; Gilfillan and Bhandari, 2022). Therefore, a comprehensive understanding of the pathogenesis of BPD will result in the development of innovative medicines for newborns at high risk.

Pyroptosis is a caspase-1/4/5/11-dependent programmed cell death that results from inflammasome activation and is accompanied by rupture of the cell membrane, pore formation, and leakage of the cell content. It is involved in the pathogenesis of respiratory diseases, such as asthma, acute lung injury, BPD, pulmonary fibrosis, and chronic obstructive pulmonary disease (Dapaah-Siakwan et al., 2019; Herrington et al., 2019; Zhang et al., 2021). In the lung tissue of newborn mice with BPD, Pan et al. discovered a remarkable increase in TNF-α, NLRP3, IL-1, and IL-6 expression as well as inflammatory infiltration, a reduction in the number and structure of alveoli, and thickening of alveolar bullae (Pan et al., 2018). Syed et al. found that hyperoxia exacerbated scorching and caused lung injury and stimulated caspase-1 activation and IL-1 secretion. In addition, it promoted the production of IL-6 and TNF-α and the activation of NLRP3 inflammatory vesicles (Syed et al., 2019). Inhibition of caspase-1 decreased the pyroptosis level, and thereby improved alveolar and vascular development in hyperoxia-exposed lungs in BPD mice (Dapaah-Siakwan et al., 2019). Simvastatin could downregulate NLRP3-related pyroptosis and attenuate lung injury in hyperoxia-induced bronchopulmonary dysplasia *via* a KLF2-mediated mechanism (Wang et al., 2022). 18β-Glycyrrhetinic acid (18β-GA) inhibited the activation of NLRP3 inflammasome and NLRP3-related pyroptosis, decreased ROS level and pulmonary inflammation, improved alveolar development of BPD rats (Qing et al., 2022). It is crucial to analyze the association between pyroptosis and BPD using several bioinformatics methods when selecting gene chip data.

This research identified a total of five different genes associated with pyroptosis, including CHMP7, NLRC4, NLRP2, NLRP6, and NLRP9. The ROC curve revealed that all five genes had AUC values higher than 0.6, indicating that the five genes can distinguish between BPD and control samples with a high degree of accuracy and specificity. The three highest AUC values were for CHMP7, NLRC4, and NLRP6. Excessive activation of CHMP7 lowers translation efficiency and may result in severe membrane deformation, in addition to affecting cell development, metabolism, cell division, and the internal environment stability (Coyne et al., 2021). Cancer cell death resistance is correlated with the activation of the CHMP7-associated ESCRT-III pathway. NLRC4 is a cytoplasmic member of the nucleotide-binding oligomerization-like receptor (NLR) family with a caspase recruitment domain (Romberg et al., 2017). Auto-inflammatory diseases are caused by NLRC4 mutations, resulting in the development of NLRC4 inflammasomes, caspase-1 activation, and IL-1 release (Karki et al., 2018). NLRP6 has a crucial role in inflammation and immunological responses of the host to the intestinal microbiota. NLRP6 also protects against the advancement of nonalcoholic fatty liver disease and obesity (Mukherjee et al., 2020; Huang et al., 2021).

Emerging evidence suggests that immune cells play a crucial role in chronic lung illness in preterm infants. TNF receptors, TNF-α, CD8^+^ T cells, and NK cells protect against viral infection and can also play a role in immunopathology due to their contact-dependent effector actions. INF-α and TNF-α are believed to be the major agents responsible for T cell-mediated lung damage (Bruder et al., 2006). Using the CIBERSORT approach, we discovered differences in various immune cells, including neutrophils, macrophages M0, macrophages M2, T cells CD4 naïve, and T cells CD8, between the control and BPD samples. Moreover, NLRP9, NLRP6, NLRP4, and CHMP7 were associated with several immune cells. These findings provide valuable references for further validation. Finally, the marker gene for the ceRNA network was analyzed. Noncoding RNA has a crucial function in the progression of BPD. There is uncertainty as to whether our projected noncoding RNA is involved, and the particular routes require further investigation. Therefore, the chosen non-coding RNA may be investigated prospectively.

Our research has some limitations. First, we discovered one of the largest BPD datasets for analysis, but the number of included samples was still small; hence, a large cohort is required to corroborate our results. There were no *in vivo* experiments and not enough *in vitro* experiments to verify the level of expression of the genes identified, thus reducing the precision of our study. Consequently, further validations are required to make the findings more convincing.

## Conclusion

Various bioinformatics approaches and online databases were used in this study. Five marker PRGs were used to establish a diagnostic nomogram for BPD. We also performed immune infiltration analysis and constructed a ceRNA network. The findings of our study may provide new insights into the molecular mechanisms of BPD.

## Data Availability

The original contributions presented in the study are included in the article/Supplementary Material, further inquiries can be directed to the corresponding authors.
