# Supplementary figures and images for: Genome-wide exploration of a pyroptosis-related gene module along with immune cell infiltration patterns in bronchopulmonary dysplasia
Source: Front Genet. 2023 Jan 4;13:1074723. doi: 10.3389/fgene.2022.1074723 (PMC9845403; doi:10.3389/fgene.2022.1074723)

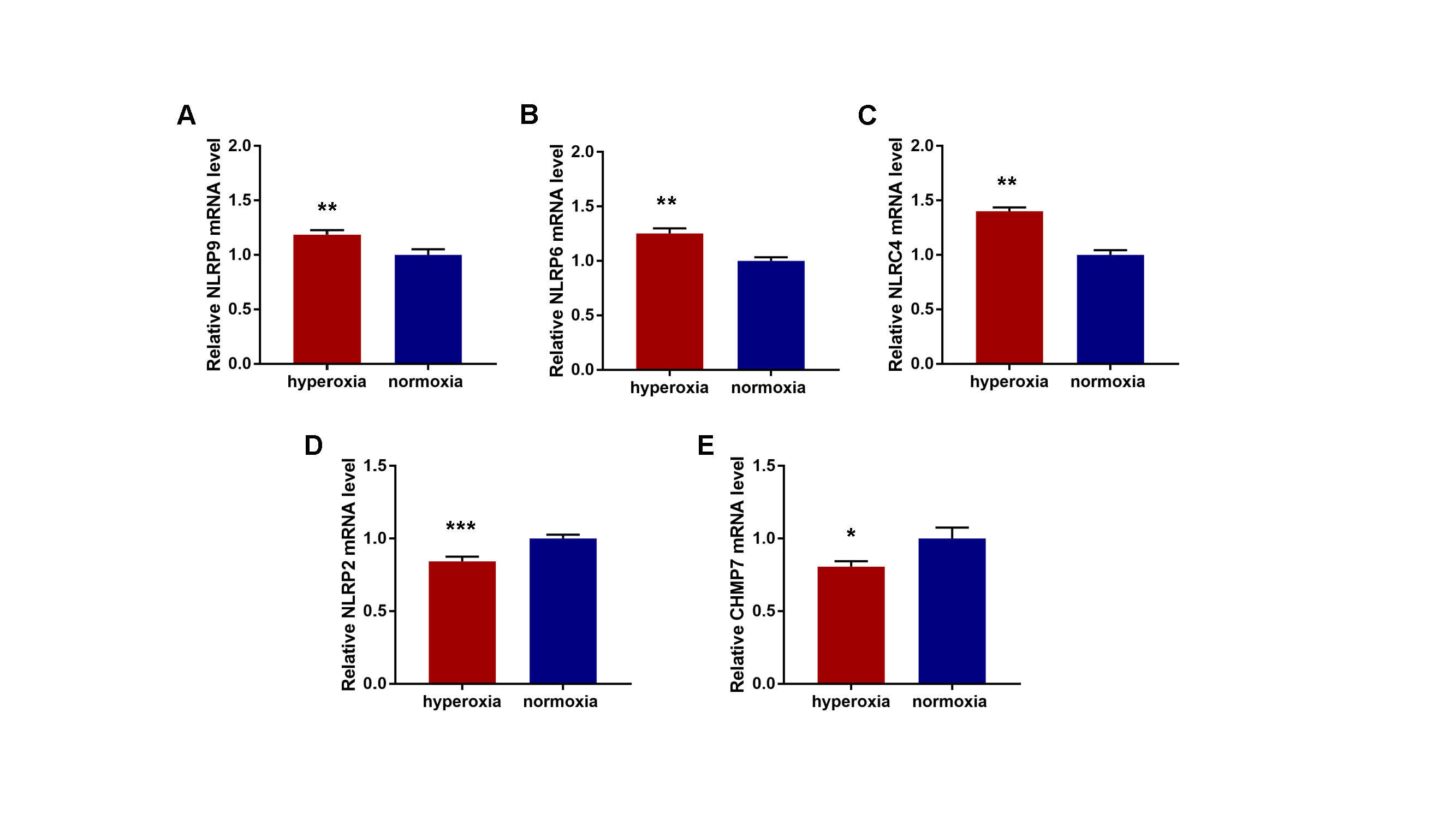

Supplement: Supplementary file 2 [file Image1.TIFF]
